# Supplementary material for: PTENα functions as an immune suppressor and promotes immune resistance in PTEN-mutant cancer
Source: Nat Commun. 2021 Aug 26;12:5147. doi: 10.1038/s41467-021-25417-6 (PMC8390757; doi:10.1038/s41467-021-25417-6)
Supplement: Supplementary file 1 — Supplementary Information [file 41467_2021_25417_MOESM1_ESM.pdf]

PTENα functions as an immune suppressor and promotes immune resistance in *PTEN*-mutant cancer

Yizhe Sun[1], Dan Lu[1*], Yue Yin[1], Jia Song[1], Yang Liu[1], Wenyan Hao[1], Fang Qi[1], Guangze Zhang[1], Xin Zhang[1], Liang Liu[1], Zhiqiang Lin[1], Hui Liang[1], Xuyang Zhao[1], Yan Jin[1], and Yuxin Yin[1,2,3*]

[1]Institute of Systems Biomedicine, Department of Pathology, School of Basic Medical Sciences, Beijing Key Laboratory of Tumor Systems Biology, Peking University Health Science Center, Beijing100191, P.R. China

[2]Peking-Tsinghua Center for Life Sciences, Peking University Health Science Center, Beijing100191, China

[3]Institute of Precision Medicine, Peking University Shenzhen Hospital, Shenzhen 518036, China

These authors contributed equally to this work: Yizhe Sun, Dan Lu.

[*]Correspondence and requests for materials should be addressed to D.L. (email: taotao@bjmu.edu.cn) or to Y.Y. (email: yinyuxin@hsc.pku.edu.cn).

Supplemental Figures:

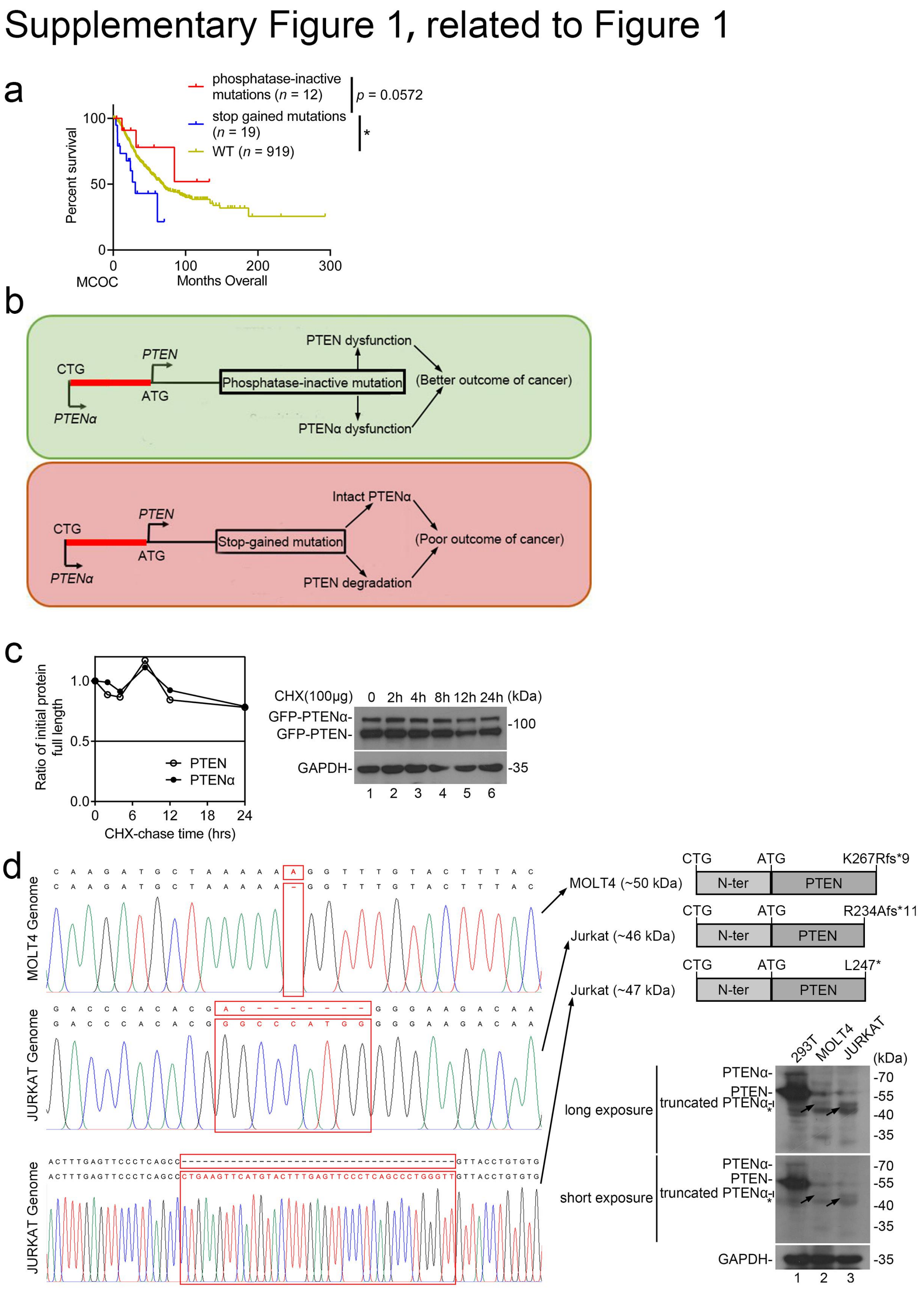

Supplementary Figure 1, related to Figure 1. PTENα remains active in *PTEN*-mutant cancer

(a) Survival curve of metastatic colorectal cancer (MCOC) patients with *PTEN* mutations (WT, n = 919; stop gained mutations, n = 19; phosphatase-inactive mutations, n = 12, *P = 0.0162). Clinical data were acquired from the Metastatic Colorectal Cancer database (MSKCC, Cancer Cell 2018[1]).

(b) Schematic diagram of the effect of *PTEN* mutations on tumor patients.

(c) HEK293T cells were transfected to express full length GFP-tagged PTEN and PTENα. The cells were treated with 100 μg/ml CHX for indicated hours. Expression of PTEN and PTENα were assessed by immunoblot analysis with anti-GFP antibody. Gray values of PTEN and PTENα relative to GAPDH were determined and used for line chart.

(d) Genomic DNA of MOLT4 or Jurkat cells were extracted, and amplificated with specific primers using PCR. Products of amplification were subjected to sequencing analysis. PTEN and PTENα in the cells were assessed by immunoblot analysis with anti-N-PTEN (EPR23729-4) antibody. Truncated PTENα in MOLT4 or Jurkat cells were indicated with the arrow. '*' refers to unspecific band.

Statistical significance was assessed by Log-rank (Mantel-Cox) test (a). Data are representative of three (c and d) independent experiments. Source data are provided as a Source Data file.

Supplementary Figure 2, related to Figure 2

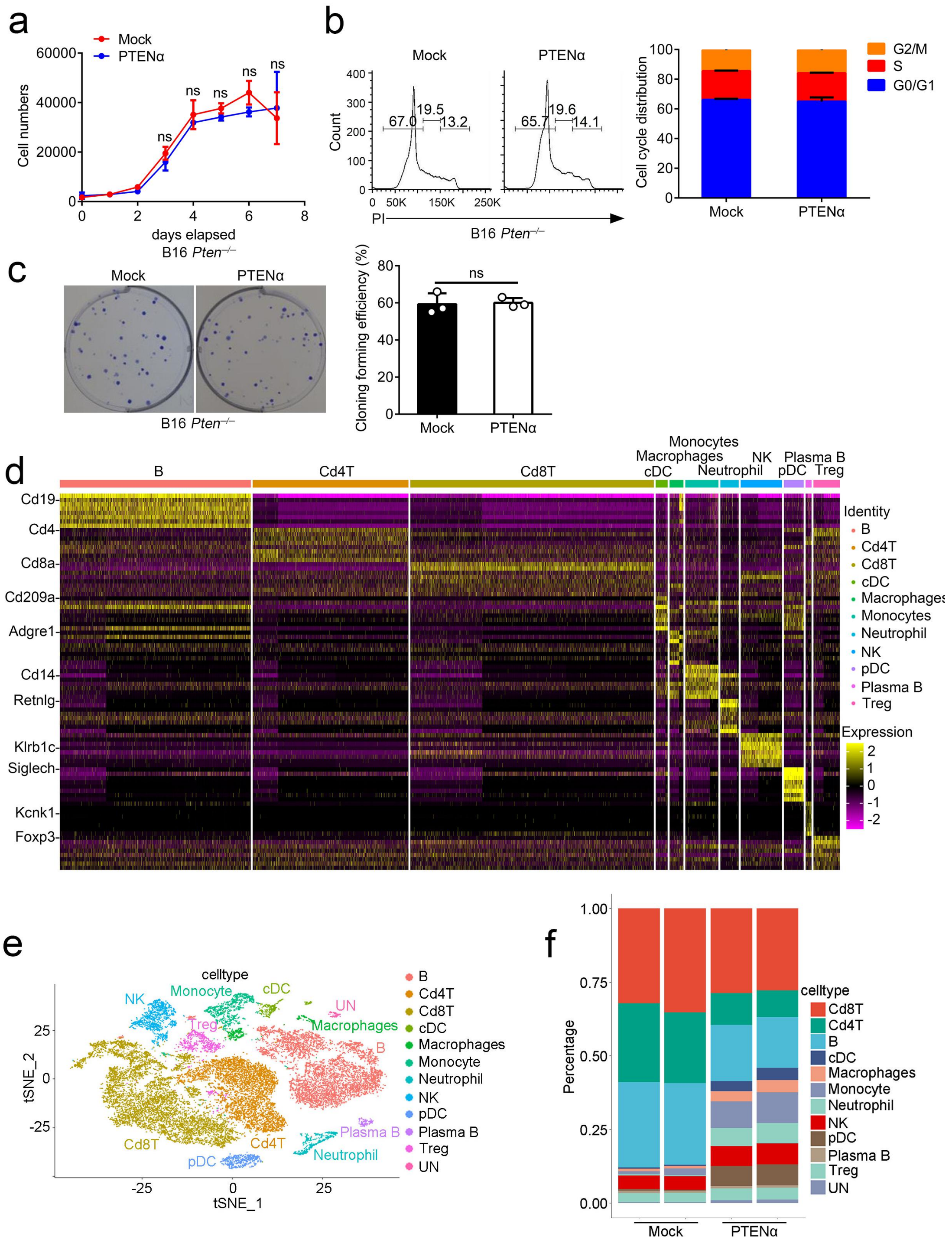

Supplementary Figure 2, related to Figure 2. PTENα diminishes the leukocyte recruitment

(a-c) *Pten*$^{-/-}$ B16 cells were transfected to express Mock or PTENα.

(a) The cell proliferation was assessed by the cell proliferation assay (n = 3 cell cultures, mean ± SD, ns, not significant).

(b) The cells were stained with PI, and subjected to flow cytometry analysis. Percentages of G0/G1, S and G2/M phase cells were used for stacked plot (n = 3 cell cultures, mean ± SD). All live cells were gated.

(c) The cells were subjected to colony formation assay, and the cloning forming efficiency were calculated by dividing the number of clones by the number of seeded cells, using for statistical analysis (n = 3 cell cultures, mean ± SD, ns, not significant).

(d-f) Mock or PTENα expressing *Pten*$^{-/-}$ B16 cells were subjected to cancer vaccine model. The tumor-infiltrated cells (TILs) were isolated when the tumor volumes of Mock group reach 200 mm^3, and the $CD45^+$ cells were sorted, using for 10x sc-RNA-seq.

(d and e) Cells were identified as 12 clusters utilizing graph-based clustering (e), and the differentially expressed genes in the clusters were used for drawing the heat map (d).

(f) Proportion of the 12 clusters were shown.

Statistical significance was assessed by two-tailed unpaired Student's t test (a,c). Data are representative of two (a-c) independent experiments. Source data are provided as a Source Data file.

Supplementary Figure 3, related to Figure 2

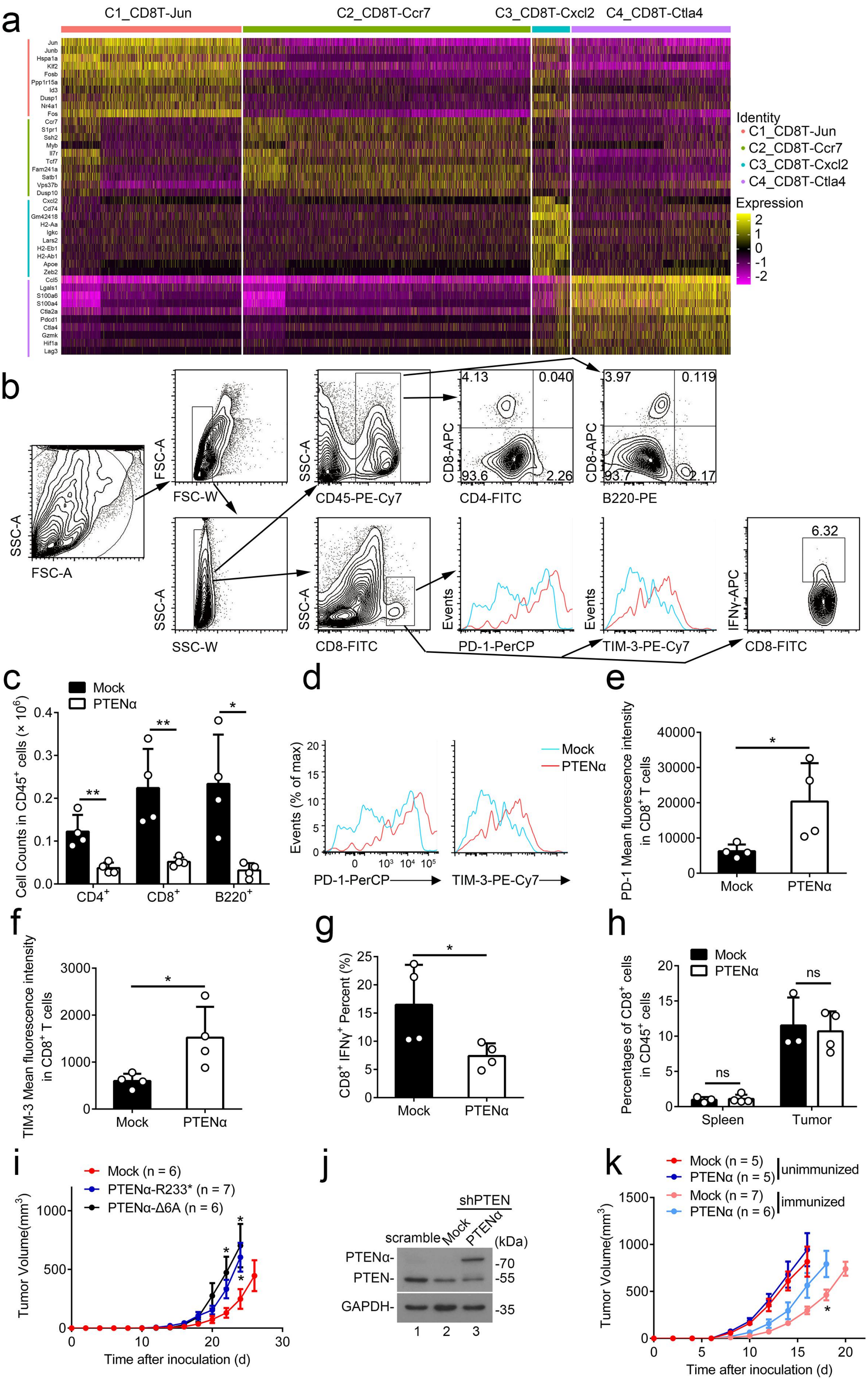

Supplementary Figure 3, related to Figure 2. PTENα promotes tumor immune

escape

(a-g) Mock or PTENα expressing *Pten*$^{-/-}$ B16 cells were subjected to cancer vaccine model. TILs were isolated when the tumor volumes of Mock-immunized group reach 200 mm^3.

(a) CD45$^+$ cells were sorted, using for 10x scRNA-seq. Cells were identified as 12 clusters utilizing graph-based clustering, and Cd8T cells were further analyzed and identified as 4 clusters utilizing graph-based clustering.

(b) Gating strategies of flow cytometry analysis of TILs.

(c-f) Cell counts of B220$^+$ cells, CD4$^+$ and CD8$^+$ T cells (c) (n = 4 mice, mean ± SD, *P = 0.0132, **P (CD4) = 0.0060, **P (CD8) = 0.0094) and the mean fluorescence intensity of PD-1 and TIM-3 in CD8$^+$ T cells (d-f) (n = 4 mice, mean ± SD, *P (PD-1) = 0.0437, *P (TIM-3) = 0.0338) were assessed by flow cytometry analysis.

(g) The lymphocytes were treated with PMA (100 ng/ml) and ionomycin (500 ng/ml) for 5 hours, and production of IFNγ in CD8$^+$ T cells was determined by flow cytometry analysis (n = 4 mice, mean ± SD, *P = 0.0499).

(h) Mock or PTENα expressing B16-*Pten*$^{-/-}$ cells were subjected to adoptive T cell transfer assay. On day 24 post tumor inoculation, immune cells were isolated from spleens and tumors of the nude mice, and subjected to flow cytometry analysis. Percentages of CD8$^+$ T cells in CD45$^+$ immune cells were shown (Mock, n = 3 mice; PTENα, n = 4 mice, mean ± SD, ns, not significant). Gating Strategy is identical to that in (b).

(i) Indicated *Pten*$^{-/-}$ B16 cells were subjected to cancer vaccination model. Tumor

volumes were monitored overtime (PTENα-R233*, n = 7 mice; other groups, n = 6 mice, mean ± s.e.m, *P (d22) = 0.0355, *P (d24, Mock vs. Δ6A) = 0.0487, *P (d24, Mock vs. R233*) = 0.0409). Statistical significances of each groups with Mock group were shown.

(j and k) PTEN expression in CT26 cells were knockdown by shRNA targeting PTEN, and then the cells were transfected with empty vector or vector encoding PTENα.

(j) Immunoblot analysis of PTENα expression in the CT26 cells with anti-PTEN (138G6) antibody.

(k) Mock or PTENα CT26-sh-PTEN cells were subjected to cancer vaccine model in Balb/c mice. The tumor volumes were monitored overtime (Unimmunized, n = 5 mice; Immunized-Mock, n = 7 mice; Immunized-PTENα, n = 6 mice, mean ± s.e.m, *P = 0.0409). Statistical significances between immunized groups were shown.

Statistical significance was assessed by two-tailed unpaired Student's t test (c,e-i,k). Data are representative of two (b-k) independent experiments. Source data are provided as a Source Data file.

Supplementary Figure 4, related to Figure 3

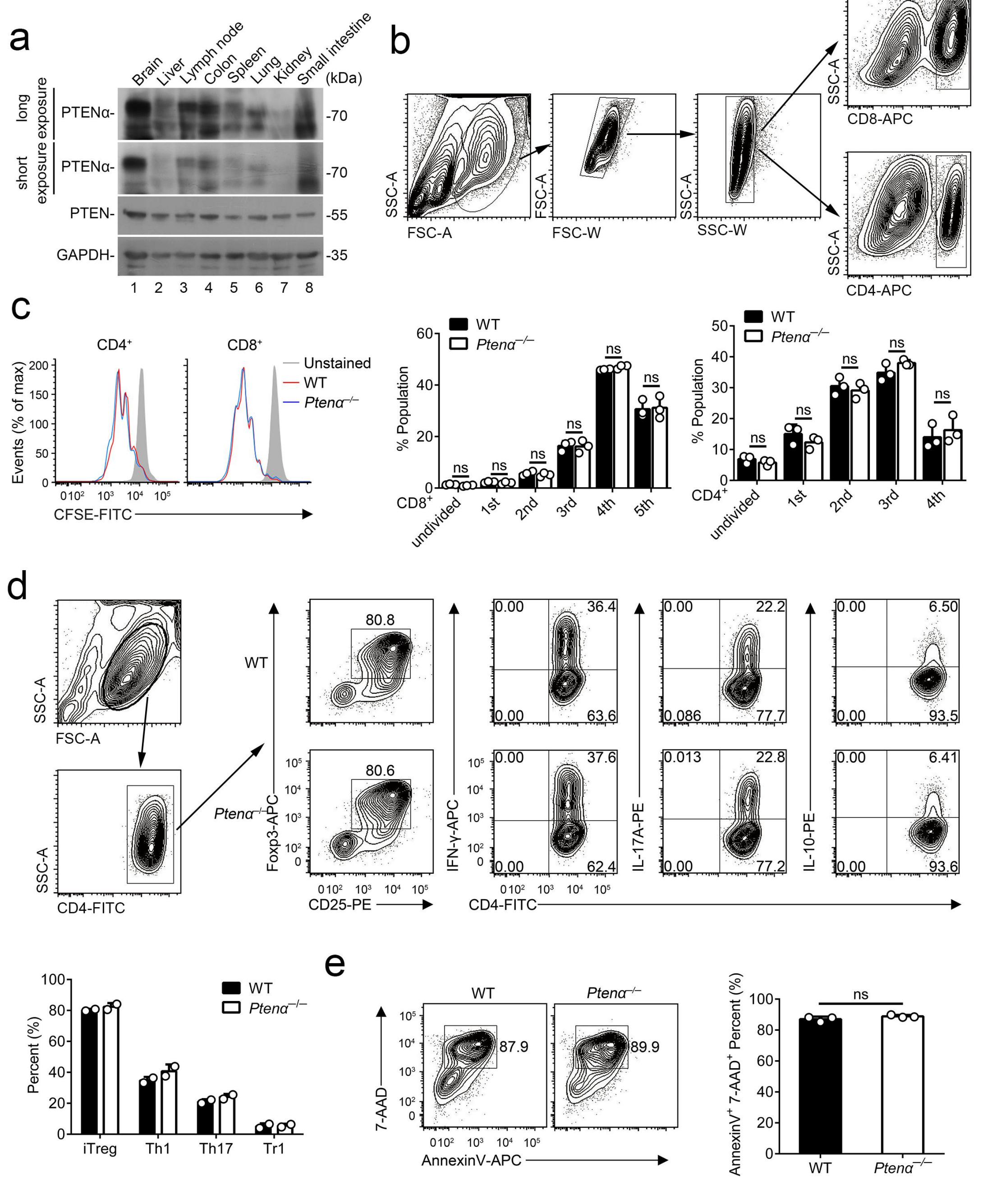

Supplementary Figure 4, related to Figure 3. PTENα hardly affects T cell proliferation, differentiation and cell death

(a) Expression profile of PTENα in C57BL/6 mice was assessed by immunoblot

analysis with anti-PTEN (138G6) antibody.

(b and c) Splenocytes from C57BL/6 mice were stained with CFSE, and activated with anti-CD3 and anti-CD28 antibodies for 60 hours. CFSE dilutions in $CD4^+$ and $CD8^+$ T cells were determined by flow cytometry analysis (c) (n = 3 mice, mean ± SD, ns, not significant). Gating strategies were shown (b).

(d) Spleens were harvested from C57BL/6 mice, and the $CD4^+$ $CD44^{low}$ cells (Naïve $CD4^+$ T cells) were sorted by a flow cell sorter, subjecting to *in vitro* differentiation assay. Percentages of iTreg ($CD4^+$ $CD25^+$ $Foxp3^+$), Th1 ($CD4^+$ $IFN\gamma^+$), Th17 ($CD4^+$ $IL\text{-}17^+$) and Tr1 ($CD4^+$ $IL\text{-}10^+$) were used for statistical analysis (n = 2 cell cultures, mean ± SD).

(e) Naïve $CD4^+$ T cells were sorted from splenocytes from C57BL/6 mice, and polarized with anti-CD3 and anti-CD28 antibodies. 72 hours post activation, cells were stained with Annexin V and 7-AAD, followed by flow cytometry analysis (n = 3 cell cultures, mean ± SD, ns, not significant). All cells were gated.

Statistical significance was assessed by two-tailed unpaired Student's t test (c and e). Data are representative of two (a-e) independent experiments. Source data are provided as a Source Data file.

Supplementary Figure 5, related to Figure 3

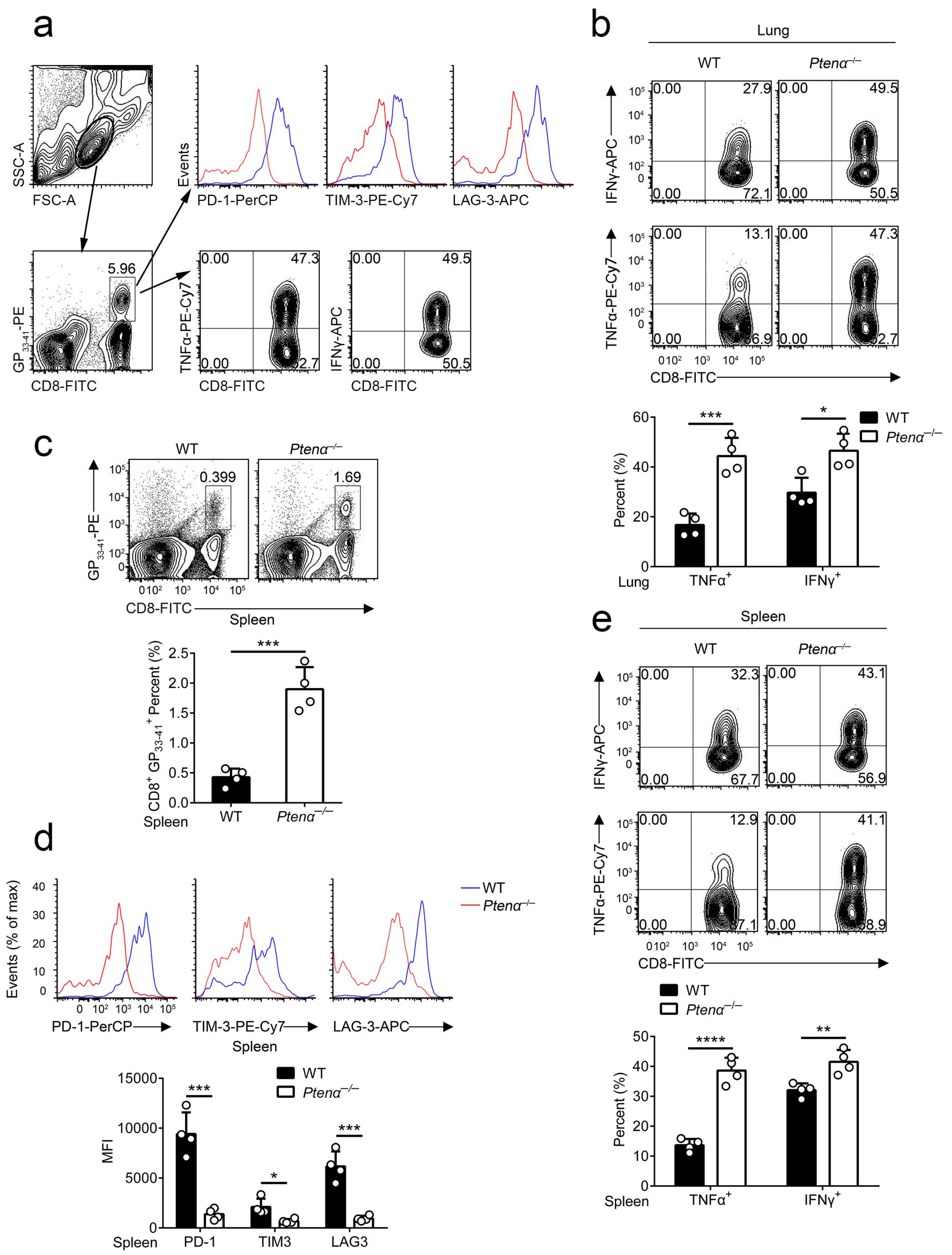

Supplementary Figure 5, related to Figure 3. Existence of PTENα promotes exhausted T cell formation

(a-e) Wild-type and *Ptenα*$^{-/-}$ mice were *i.v.* infected with 5×10^5 PFU LCMV-Cl13.

On day 30 post infection, lymphocytes were isolated from lungs or spleens of the mice, and subjected to flow cytometry analysis.

(a) Gating strategies of the flow cytometry analysis.

(b) Lymphocytes from lungs of the mice were activated by 2 μg/ml $GP_{33\text{-}41}$ peptides. Flow cytometry was used to analyze the expression profiles of TNF-α and IFN-γ on $CD8^+$ $GP_{33\text{-}41}{}^+$ cells (n = 4 mice, mean ± SD, *P = 0.0103, ***P = 0.0007).

(c) Lymphocytes from spleens were subjected to flow cytometry analysis. Percentages of $CD8^+$ $GP_{33\text{-}41}{}^+$ cells were used for statistical analysis (n = 4 mice, mean ± SD, ***P = 0.0003).

(d) Expression levels of PD-1, TIM-3 and LAG-3 on the $CD8^+$ $GP_{33\text{-}41}{}^+$ cells isolated from the spleens of the infected mice were determined by flow cytometry analysis (n = 4 mice, mean ± SD, *P = 0.0165, ***P (PD-1) = 0.0004, ***P (LAG-3) = 0.0005).

(e) The lymphocytes from spleens were treated with $GP_{33\text{-}41}$ peptide for 5 hours. Production of TNFα and IFNγ of the $CD8^+$ $GP_{33\text{-}41}{}^+$ cells were determined by flow cytometry analysis (n = 4 mice, mean ± SD, **P = 0.0061, ****P < 0.0001).

Statistical significance was assessed by two-tailed unpaired Student's t test (b-e). Data are representative of two (a-e) independent experiments. Source data are provided as a Source Data file.

Supplementary Figure 6, related to Figure 3

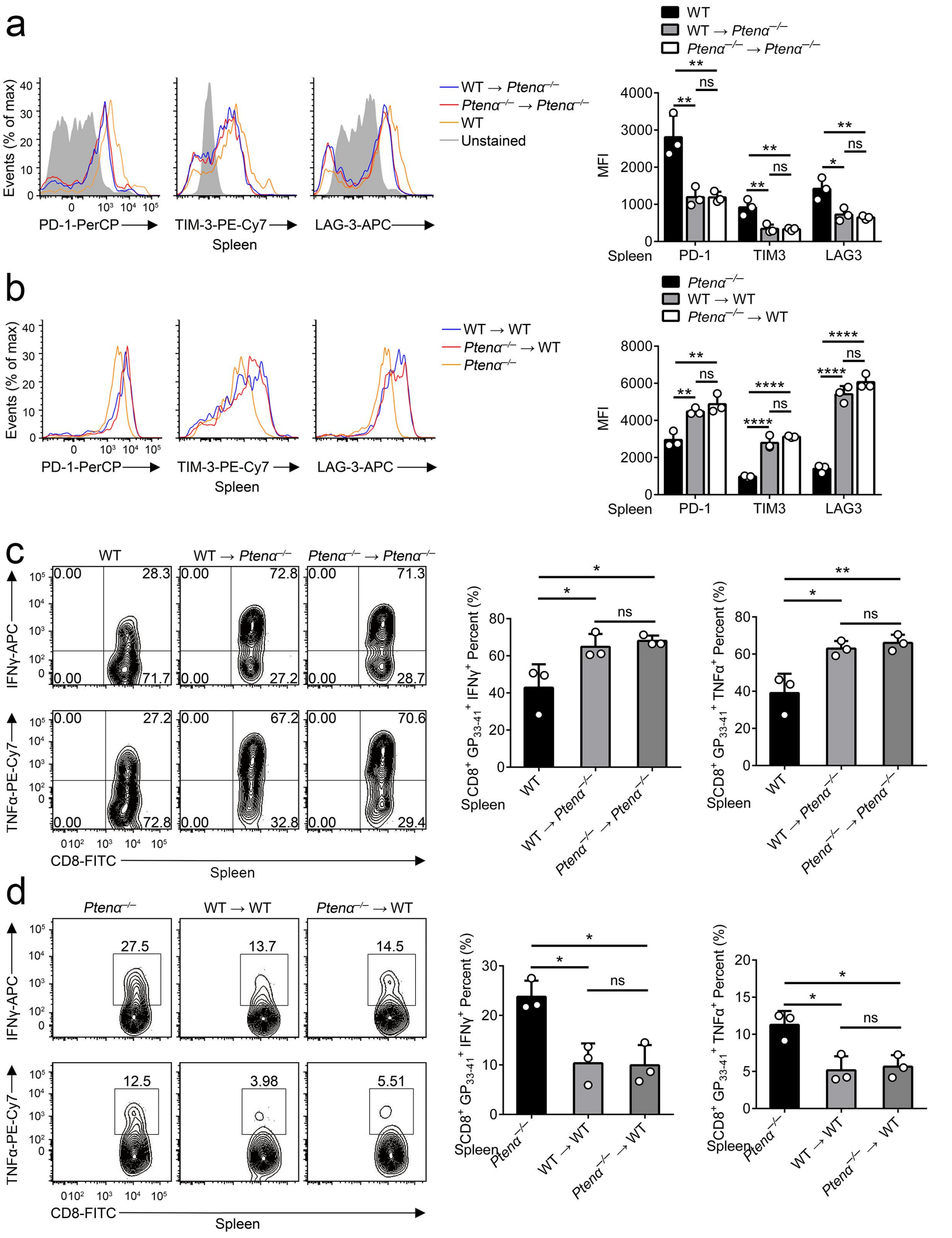

Supplementary Figure 6, related to Figure 3. T cell-extrinsic PTENα drives T cell exhaustion

Bone marrow from wild-type and *Ptenα*−/−mice were transplanted into *Ptenα*−/− (a

and c) or wild-type mice (b and d), respectively. 30 days after transplantation, mice were *i.v.* infected with 5 × 10^5 PFU LCMV-Cl13. On day 30 post infection, spleens were harvested from the mice, and lymphocytes were isolated. Expression levels of PD-1, TIM-3 and LAG-3 on the $CD8^+$ GP_{33-41}^+ cells were determined by flow cytometry analysis (a and b) (n = 3 mice, mean ± SD, ns, not significant, *$P = 0.0129$, **$P < 0.01$, ****$P < 0.0001$). The splenocytes were treated with GP_{33-41} peptide for 5 hours, and production of TNFα and IFNγ of the $CD8^+$ GP_{33-41}^+ cells were assessed by flow cytometry analysis (c and d) (n = 3 mice, mean ± SD, ns, not significant, *$P < 0.05$, **$P = 0.0073$). Gating strategies were identical to that in Supplementary Figure 5a.

Statistical significance was assessed by one-way ANOVA followed by Tukey's multiple comparisons test (a-d). Data are representative of two (a-d) independent experiments. Source data are provided as a Source Data file.

Supplementary Figure 7, related to Figure 4

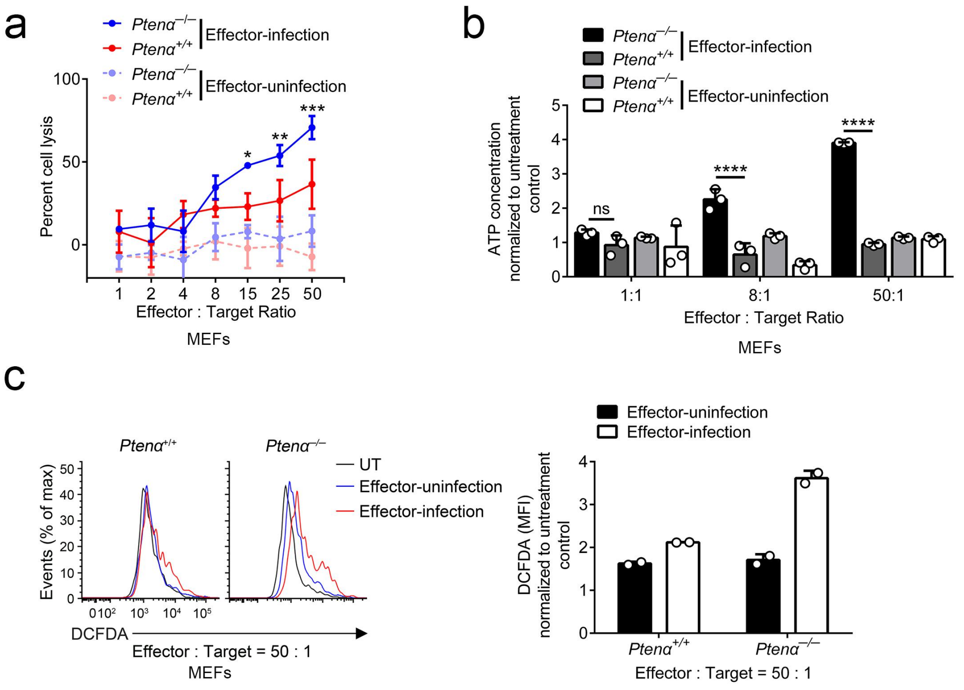

Supplementary Figure 7, related to Figure 4. PTENα protects cells from T cell cytotoxicity

(a-c) $CD8^+$ T cells were isolated from C57BL/6 mice *i.v.* infected with 1×10^6 LCMV-Cl13 for 7 days, using as effector cells for *in vitro* cytotoxicity assay. *Ptenα*$^{+/+}$ and *Ptenα*$^{-/-}$ MEFs were pulsed with $GP_{33\text{-}41}$ peptide and used as target cells. Effector cells and target cells were incubated at indicated ratios.

(a) 20 hours post incubation, MEFs cells were washed to remove lymphocytes, and counted using CCK-8 (n = 3 cell cultures, mean ± SD, **P* = 0.0135, ***P* = 0.0056, ****P* = 0.0003).

(b) Culture medium was collected 20 hours post incubation, and centrifuged to remove cells and debris. The supernatant was subjected to ATP content assay (n = 3 cell cultures, mean ± SD, ns, not significant, *****P* < 0.0001). Untreatment control

refers to pulsed MEFs without effector incubation.

(c) 8 hours post incubation, MEFs were harvested and stained with DCFDA, using for flow cytometry analysis. Gating strategy was identical to that in Figure 4h. Mean fluorescence intensity (MFI) of DCFDA was used for statistical analysis (n = 2 cell cultures, mean ± SD). UT, untreatment. Untreatment control refers to pulsed MEFs without effector incubation.

Statistical significance was assessed by two-way ANOVA followed by Tukey's multiple comparisons test (a and b). Data are representative of two (a-c) independent experiments. Source data are provided as a Source Data file.

Supplementary Figure 8, related to Figure 5

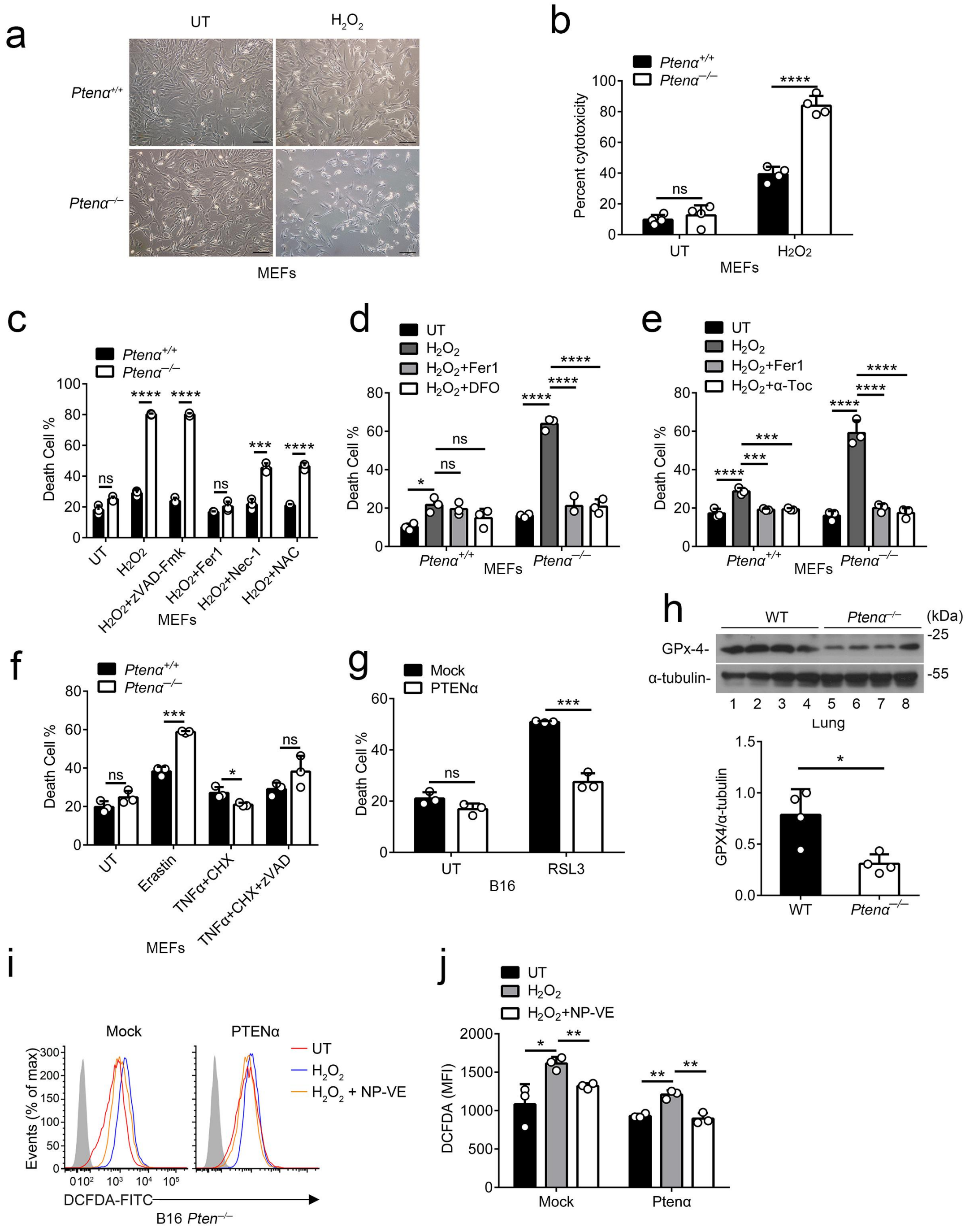

Supplementary Figure 8, related to Figure 5. PTENα blocks ferroptotic cell death

(a) Microscopy analysis of H_2O_2 (500 μM) treated *Ptenα*$^{+/+}$ and *Ptenα*$^{-/-}$ MEFs. UT,

untreatment. Scale bar: 100μm.

(b) *Ptena*$^{+/+}$ and *Ptena*$^{-/-}$ MEFs were treated with 500 μM H_2O_2, and the percent cytotoxicity was assessed using LDH release assay (n = 4 cell cultures, mean ± SD, ns, not significant, ****$P < 0.0001$). UT, untreatment.

(c) *Ptena*$^{+/+}$ and *Ptena*$^{-/-}$ MEFs were treated with 500 μM H_2O_2 in the absence or presence of various cell death inhibitors, including z-VAD-Fmk (20 μM) (apoptosis inhibitor), Fer1 (1 μM) (ferroptosis inhibitor), Nec-1 (50 μM) (necroptosis inhibitor) and NAC (1 mM) (ROS scavenger). Cells were stained with propidiumiodide (PI), followed by flow cytometric analysis. The death cell rates were used for statistical analysis (n = 3 cell cultures, mean ± SD, ns, not significant, ***$P = 0.0008$, ****$P < 0.0001$). All cells were gated. UT, untreatment.

(d) Flow cytometry analysis of cell death rates of *Ptena*$^{+/+}$ and *Ptena*$^{-/-}$ MEFs treated with 500 μM H_2O_2 plus ferroptosis inhibitor including Fer1 (1 μM) or DFO (100 μM) using propidiumiodide (PI) staining (n = 3 cell cultures, mean ± SD, ns, not significant, *$P = 0.0174$, ****$P < 0.0001$). All cells were gated. UT, untreatment.

(e) *Ptena*$^{+/+}$ and *Ptena*$^{-/-}$ MEFs were treated with 500 μM H_2O_2 plus ferroptosis inhibitor including Fer1 (1 μM) or α-Toc (100 μM), followed by assessing with propidiumiodide (PI) staining (n = 3 cell cultures, mean ± SD, ***P (Fer1) = 0.0003, ***P (α-Toc) = 0.0004, ****$P < 0.0001$). All cells were gated. UT, untreatment.

(f) *Ptena*$^{+/+}$ and *Ptena*$^{-/-}$ MEFs were treated with Erastin (5 μM) to induce ferroptosis. Apoptotic and necroptotic cell death were induced with 100 ng/ml TNFα and 2 μg/ml CHX in the absence or presence of z-VAD-Fmk (25 μM), respectively. The results

were analyzed using propidiumiodide (PI) staining, followed by flow cytometry analysis. Death cell rates were used for statistical analysis (n = 3 cell cultures, mean ± SD, ns, not significant, *P = 0.0285, ***P = 0.0001). All cells were gated. UT, untreatment.

(g) Mock or PTENα expressing *B16-Pten*$^{-/-}$ cells were treated with 1 μM RSL3 for 12 hours, and stained with PI. The cells were subjected to flow cytometry analysis, and the death cell rates were used for statistical analysis (n = 3 cell cultures, mean ± SD, ***P = 0.0003). All cells were gated. UT, untreatment.

(h) Wild-type and *Ptenα*$^{-/-}$ mice were *i.v.* infected with LCMV-Cl13. On day 7 post infection, lungs were harvested, and subjected to immunoblot analysis with anti-GPX4 antibody. Gray values of GPX4 were determined and used for statistical analysis (n = 4 mice, mean ± SD, *P = 0.0114).

(i and j) Mock or PTENα expressing *Pten*$^{-/-}$ B16 cells were treated with 100 μM H_2O_2 in the presence or absence of 20mM NP-VE for 1 hour, followed by staining with DCFDA. Mean fluorescence intensities of DCFDA were determined by flow cytometry analysis (i), and used for statistical analysis (j) (n = 3 cell cultures, mean ± SD, *P < 0.05, **P (Mock) = 0.0056, **P (PTENα, UT vs. H_2O_2) = 0.0015, **P (PTENα, H_2O_2 vs. NPVE) = 0.0038). All live cells were gated for flow cytometry analysis.

Statistical significance was assessed by two-tailed unpaired Student's t test (b,c,f-h,j) or one-way ANOVA followed by Tukey's multiple comparisons test (d and e). Data are representative of two (a-j) independent experiments. Source data are provided as a

Source Data file.

Supplementary Figure 9, related to Figure 5

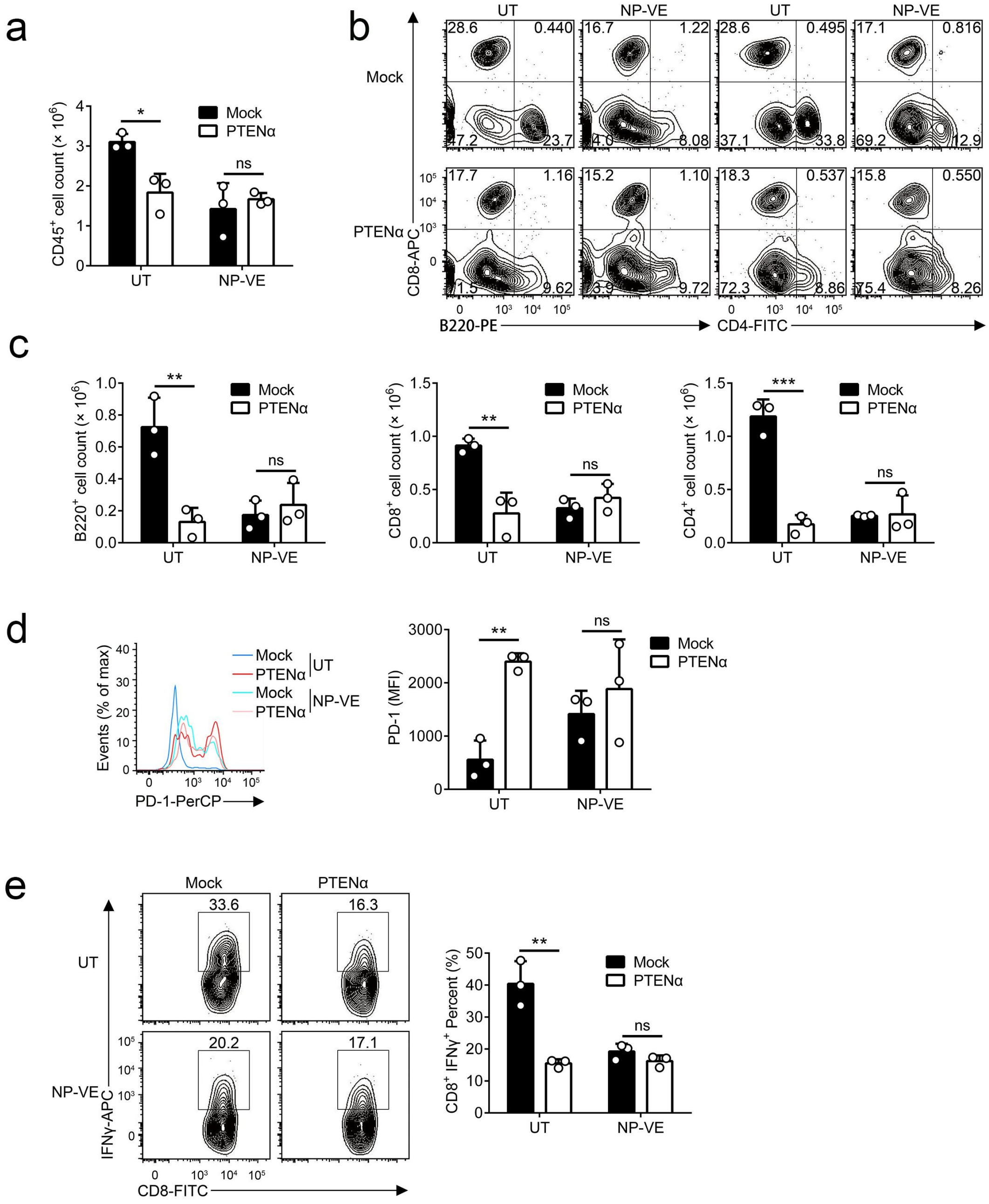

Supplementary Figure 9, related to Figure 5. Reduction of immunogenic cell death is essential for PTENα in promotion of T cell dysfunction

(a-e) Mock or PTENα transfected *Pten*$^{-/-}$ B16 cells were subjected to cancer vaccination model. The cells were subcutaneously injected in the presence or absence of 50 µl of 5 M NP-VE. Tumors were collected when the Mock-UT group reached

200 mm^3, and tumor infiltrating immune cells were isolated and subjected to flow cytometry analysis. Gating strategies were identical to that in Supplementary Figure 3b. UT refers to untreatment.

(a) The cell counts of $CD45^+$ cells were used for statistical analysis (n = 3 mice, mean ± SD, ns, not significant, *P = 0.0128).

(b and c) Cell counts of $B220^+$ cells, $CD4^+$ and $CD8^+$ T cells were assessed by flow cytometry analysis (n = 3 mice, mean ± SD, ns, not significant, **P (B220) = 0.0073, **P (CD8) = 0.0057, ***P = 0.0006).

(d) The mean fluorescence intensities of PD-1 in $CD8^+$ T cells were determined by flow cytometry analysis (n = 3 mice, mean ± SD, ns, not significant, **P = 0.0013).

(e) The cells were treated with PMA and ionomycin for 5 hours, and production of IFNγ in $CD8^+$ T cells were assessed by flow cytometry analysis (n = 3 mice, mean ± SD, ns, not significant, **P = 0.0039).

Statistical significance was assessed by two-tailed unpaired Student's t test (a,c-e). Data are representative of two (a-e) independent experiments. Source data are provided as a Source Data file.

Supplementary Figure 10, related to Figure 7

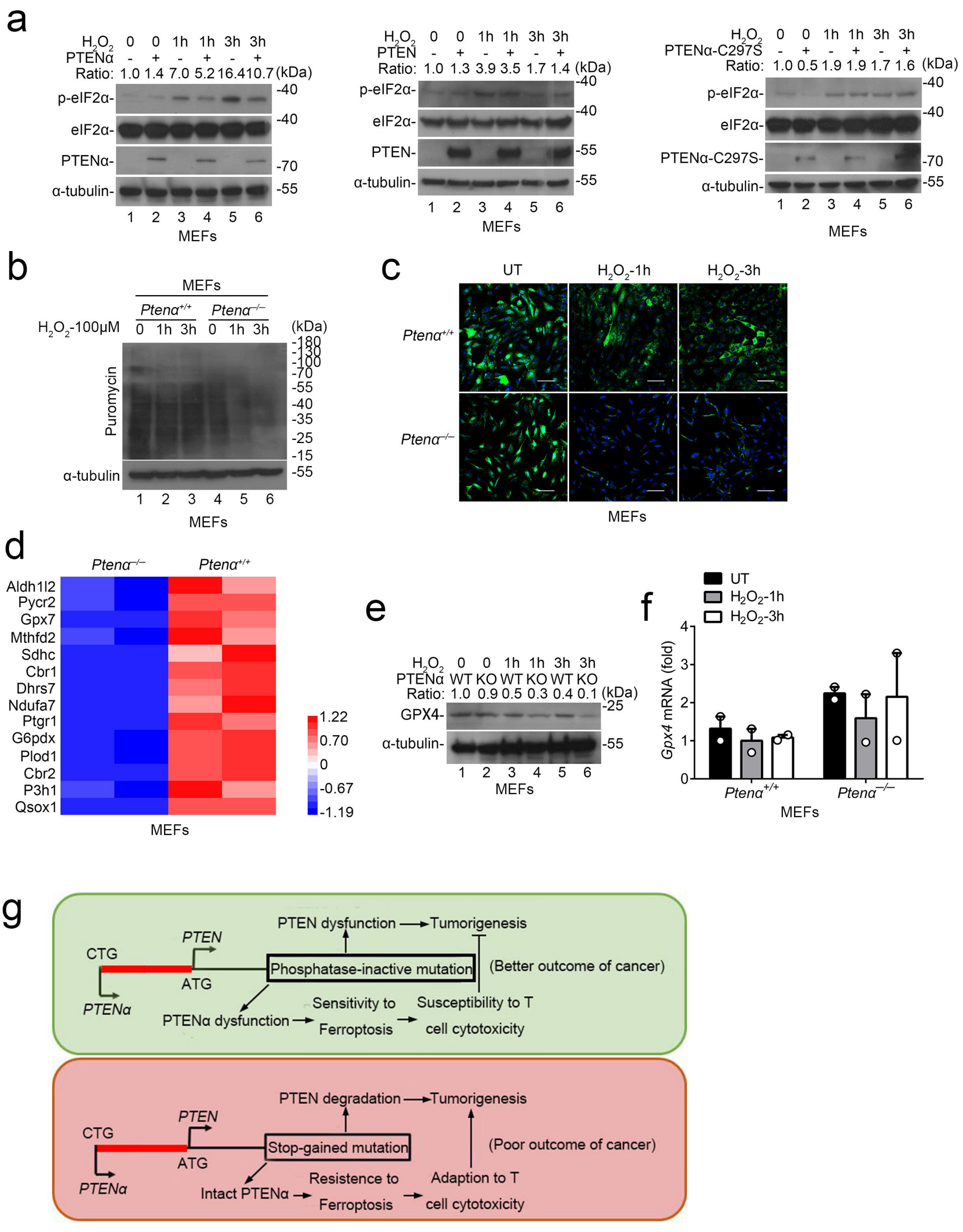

Supplementary Figure 10, related to Figure 7. PTENα limits eIF2α phosphorylation-mediated translational shutdown

(a) *Pten*$^{-/-}$ MEFs were transduced to express FLAG-tagged PTEN, PTENα or

PTENα-C297S (phosphatase-dead PTENα mutant). The cells were treated with 100 μM H_2O_2 for indicated hours, followed by immunoblot analysis with anti-eIF2α and anti-phospho-eIF2α antibodies. Gray values of total eIF2α and phosphorylated eIF2α were determined, and the ratios of total eIF2α to phosphorylated eIF2α were indicated.

(b and c) $Ptenα^{+/+}$ and $Ptenα^{-/-}$ MEFs were treated with 100 μM H_2O_2 for indicated hours, and subjected to puromycin incorporation assay. Translational efficiency was assessed by identification of the puromycin-labeled proteins using immunoblot analysis (b) and confocal fluorescence microscopy (c). Scale bars, 100 μm. UT, untreatment.

(d) $Ptenα^{+/+}$ and $Ptenα^{-/-}$ MEFs were treated with 100 μM H_2O_2 for 24 hours, followed by mass spectrometry analysis. Proteins that were downregulated in $Ptenα^{-/-}$ cells were subjected to GO terms analysis. The heat map presents proteins that are enriched in oxidation-reduction process using normalized protein expression levels.

(e and f) Immunoblot analysis (e) and qRT-PCR analysis (f) (n = 2 cell cultures, mean ± SD) of GPX4 protein and mRNA expression level in $Ptenα^{+/+}$ and $Ptenα^{-/-}$ MEFs treated with 100 μM H_2O_2 for indicated hours. Gray values of Gpx4 and GAPDH were determined, and ratio of Gpx4 to GAPDH is indicated. UT, untreatment.

(g) Schematic diagram of the role of PTENα in tumor.

Data are representative of two (a-c,e,f) independent experiments. Source data are provided as a Source Data file.

Supplemental tables

Supplementary Table 1: List of marker genes of the clusters in scRNA-seq

cell_type	Marker gene							
B	*Cd79a*	*Cd19*	*Cd79b*	*Ms4a1*	*Fcer2a*	*Fcmr*	*Cd22*	*Ighd*
Cd4T	*Cd4*	*Cd5*	*Cd40lg*	*Tmem64*	*Id3*	*S1pr1*	*Ndrg3*	*Dusp10*
Cd8T	*Cd8a*	*Cd8b1*	*Mxd4*	*Sh2d2a*	*Cd226*	*Tubb5*	*Atad2*	*Tagap*
cDC	*Cd209a*	*Kdm6b*	*H2-DMa*	*Ppt1*	*Itgax*	*Naga*	*Cd209d*	*H2-DMb1*
Macrophages	*Adgre1*	*Blvrb*	*C1qc*	*Neil1*	*Mcm3*	*Ccl8*	*Hells*	*C1qa*
Monocyte	*Fn1*	*Apoc2*	*Cd14*	*C3*	*Pgam1*	*Bach1*	*Csf1r*	*Lrp1*
Neu	*Retnlg*	*S100a9*	*Stfa2l1*	*Dedd2*	*Nktr*	*Npepps*	*Arg2*	*S100a8*
NK	*Ptprc*	*Ncr1*	*Klrk1*	*Klrb1c*	*Gzmb*	*Prf1*	*Klra8*	*Klra4*
pDC	*Siglech*	*Bst2*	*Cox6a2*	*Klk1*	*Sh3bgr*	*Gm21762*	*Lrp8*	*Cd300c*
Plasma B	*Sdc1*	*Il17re*	*Kcnk1*	*Cryba4*	*Sox13*	*Il23r*	*Il17a*	*Actn2*
Treg	*Foxp3*	*Ctla4*	*Hif1a*	*Arl5a*	*Ltb*	*Tmem65*	*Tspan32*	*Slamf1*

Supplementary Table 2: List of primers for qRT-PCR analysis in this study.

**Gene name**	**Forward primer (5'->3')**	**Reverse primer (5'->3')**
Actb	GGCTGTATTCCCCTCCATCG	CCAGTTGGTAACAATGCCATGT
Gpx4	GATGGAGCCCATTCCTGAACC	CCCTGTACTTATCCAGGCAGA
LCMV-GP	TGCCTGACCAAATGGATGATT	CTGCTGTGTTCCCGAAACACT

References

1. Yaeger, R. *et al.* Clinical Sequencing Defines the Genomic Landscape of Metastatic Colorectal Cancer. *Cancer cell* **33**, 125-136.e123 (2018).
