# Supplementary material for: PTENα functions as an immune suppressor and promotes immune resistance in PTEN-mutant cancer
Source: Nat Commun. 2021 Aug 26;12:5147. doi: 10.1038/s41467-021-25417-6 (PMC8390757; doi:10.1038/s41467-021-25417-6)
Supplement: Supplementary file 2 — Reporting Summary [file 41467_2021_25417_MOESM2_ESM.pdf]

nature research

Corresponding author(s): Yuxin Yin

Last updated by author(s): Aug 4, 2021

Reporting Summary

Nature Research wishes to improve the reproducibility of the work that we publish. This form provides structure for consistency and transparency in reporting. For further information on Nature Research policies, see our Editorial Policies and the Editorial Policy Checklist.

Statistics

For all statistical analyses, confirm that the following items are present in the figure legend, table legend, main text, or Methods section.

n/a	Confirmed	
☐	☒	The exact sample size (*n*) for each experimental group/condition, given as a discrete number and unit of measurement
☐	☒	A statement on whether measurements were taken from distinct samples or whether the same sample was measured repeatedly
☐	☒	The statistical test(s) used AND whether they are one- or two-sided<br>*Only common tests should be described solely by name; describe more complex techniques in the Methods section.*
☒	☐	A description of all covariates tested
☐	☒	A description of any assumptions or corrections, such as tests of normality and adjustment for multiple comparisons
☐	☒	A full description of the statistical parameters including central tendency (e.g. means) or other basic estimates (e.g. regression coefficient) AND variation (e.g. standard deviation) or associated estimates of uncertainty (e.g. confidence intervals)
☐	☒	For null hypothesis testing, the test statistic (e.g. *F*, *t*, *r*) with confidence intervals, effect sizes, degrees of freedom and *P* value noted<br>*Give P values as exact values whenever suitable.*
☒	☐	For Bayesian analysis, information on the choice of priors and Markov chain Monte Carlo settings
☒	☐	For hierarchical and complex designs, identification of the appropriate level for tests and full reporting of outcomes
☐	☒	Estimates of effect sizes (e.g. Cohen's *d*, Pearson's *r*), indicating how they were calculated

Our web collection on statistics for biologists contains articles on many of the points above.

Software and code

Policy information about availability of computer code

Data collection	Flow cytometry: FACSuite Software Bundle v1.0 (BD Biosciences) and FACSDiva Software v6.1 (BD Biosciences).<br>Confocal microscopy: NIS-Elements AR Analysis 4.20.00 64bit (Nikon)<br>Quantitative real-time PCR: 7500 Software v2.3 (Applied Biosystems)
Data analysis	GraphPad Prism v6.01 was used for statistical analyses.<br>NIS Elements Viewer 4.20 was used to analyze the confocal microscopy data.<br>FlowJo v7.6.1 was used for the flow cytometry data.<br>GSEA software from Broad Institute 21 (http://www.broad.mit.edu/gsea/software/software_index.html) was used to perform gene-set enrichment analysis.<br>Cell Ranger (version 6.0.1), R package Seurat (version 4.0.1), R package dplyr (1.0.6) and R package ggplot2 (3.3.3) were used to analyze the scRNA-seq data.

For manuscripts utilizing custom algorithms or software that are central to the research but not yet described in published literature, software must be made available to editors and reviewers. We strongly encourage code deposition in a community repository (e.g. GitHub). See the Nature Research guidelines for submitting code & software for further information.

Data

Policy information about availability of data

All manuscripts must include a data availability statement. This statement should provide the following information, where applicable:

- Accession codes, unique identifiers, or web links for publicly available datasets
- A list of figures that have associated raw data
- A description of any restrictions on data availability

The list of figures that have associated source data is provided. The mass spectrometry proteome data generated in this study have been deposited in the Integrated Proteome Resources (iProX), an official member of ProteomeXchange Consortium, with the accession number IPX0002523000 (https://www.iprox.cn//page/project.html?id=IPX0002523000) and IPX0003269000 (https://www.iprox.cn//page/project.html?id=IPX0003269000). The scRNAseq data have been deposited in the GEO database with the accession code GSE178258 (https://www.ncbi.nlm.nih.gov/geo/query/acc.cgi?acc=GSE178258). Sequencing data of PTEN in Molt4 and Jurkat cells have been deposited in the NCBI Genbank nucleotide database under accession code MZ615337 (https://www.ncbi.nlm.nih.gov/nuccore/MZ615337), MZ615338 (https://www.ncbi.nlm.nih.gov/nuccore/MZ615338) and MZ615339 (https://www.ncbi.nlm.nih.gov/nuccore/MZ615339). Phosphatase-inactive PTEN mutations are from Uniprot database (https://www.uniprot.org/uniprot/P60484). Clinical data of the tumor patients carrying PTEN mutations from the TCGA (UCEC, PanCancer Atlas) and Metastatic Colorectal Cancer databases (MSKCC, Cancer Cell 201833) can be acquired from cBioPortal (https://www.cbioportal.org). GO gene-sets are from MSigDB (http://www.gsea-msigdb.org/gsea/downloads.jsp). The remaining data are available within the Article, Supplementary Information or Source Data file.

Field-specific reporting

Please select the one below that is the best fit for your research. If you are not sure, read the appropriate sections before making your selection.

☒ Life sciences ☐ Behavioural & social sciences ☐ Ecological, evolutionary & environmental sciences

For a reference copy of the document with all sections, see nature.com/documents/nr-reporting-summary-flat.pdf

Life sciences study design

All studies must disclose on these points even when the disclosure is negative.

Sample size	No statistical methods were used to predetermine the sample size. Sample size was chosen based on previous experience by our group on similar experiments (PMID 26479789 and 31932812).
Data exclusions	No data were excluded from the analysis.
Replication	All experiments were independently replicated at least two times and similar results were generated.
Randomization	For animal studies using wild-type and Ptenα–/– mice, no randomization was performed, and samples were grouped by genotypes. Other animals or cells were measured at random within each condition.
Blinding	Data acquisition and analyses were not blinded but all assays were performed at the same time for all groups of a given experiment. Since all conditions were subjected to the same analyses, blinding was not considered to be necessary.

Reporting for specific materials, systems and methods

We require information from authors about some types of materials, experimental systems and methods used in many studies. Here, indicate whether each material, system or method listed is relevant to your study. If you are not sure if a list item applies to your research, read the appropriate section before selecting a response.

Materials & experimental systems

n/a	Involved in the study	
☐	☒	Antibodies
☐	☒	Eukaryotic cell lines
☒	☐	Palaeontology and archaeology
☐	☒	Animals and other organisms
☒	☐	Human research participants
☒	☐	Clinical data
☒	☐	Dual use research of concern

Methods

n/a	Involved in the study	
☒	☐	ChIP-seq
☐	☒	Flow cytometry
☒	☐	MRI-based neuroimaging

Antibodies

Antibodies used	Following commercial antibodies were used in this study. clone number, catalogue number and dilutions were shown in turn: anti-PTEN (138G6, 9559, 1:1000), anti-eIF2α (D7D3, 5324, 1:1000) and antibody to phosphorylated eIF2α (Ser51) (119A11, 3597, 1:1000)

(all from CST); anti-GAPDH (1C4, KM9002T, 1:5000) and anti-GFP (9F6, KM8009, 1:5000) (both from Sungenebiotech); anti-FLAG (M2, F3165, 1:5000, Sigma-Aldrich); anti-α-tubulin (2F9, M175-3, 1:5000), H-2Db LCMV gp33 Tetramer-KAVYNFATC (TB-5002-1, 1:250) (both from MBL); anti-puromycin (17H1, MABE341, 1:1000, merck-millipore); anti-GPX4 (E-12, sc-166570, 1:1000) and anti-LCMV (M104, sc-57894, 1:100) (all from Santa Cruz); anti-Hsp90 (OTI4C10, TA-12, 1:1000, ZSGB-BIO); anti-N-PTEN (EPR23729-4, ab260011, 1:1000), anti-HMGB1 (EPR3507, ab79823, 1:1000) and anti-Hsp70 (EPR16892, ab181606, 1:1000) (all from abcam); anti-IL-17A (eBio17B7, 12-7177-81, 1:250), anti-IL-10 (JES5-16E3, 12-7101-41, 1:250), anti-Foxp3 (FJK-16s, 17-5773-82, 1:250), anti-PD-1 (J43, 46-9985-82, 1:250), anti-TIM3 (RMT3-23, 25-5870-82, 1:250), anti-LAG3 (C9B7W, 17-2231-82, 1:250), anti-CD45 (30-F11, 25-0451-81, 1:250), anti-CD25 (PC61.5, 12-0251-83, 1:250), antibody to IFN-γ (XMG1.2, 17-7311-82, 1:250) and antibody to TNF-α (MP6-XT22, 25-7321-82, 1:250) (all from eBioscience); Anti-CD4 (GK1.5, 35-0041-U500, 1:250) and anti-CD8 (53-6.7, 35-0081-U500 and 20-0081-U100, 1:250) (both from Tonbo); anti-G3BP1 (A14836, 1:1000, ABclonal); Anti-CD3ε (145-2C11, 100301, 2 μg/ml), anti-CD28 (37.51, 102101, 1 μg/ml), anti-IL-4 (11B11, 504101, 10 μg/ml) and anti-IFNγ (XMG1.2, 505801, 10 μg/ml) (all from BioLegend).

Validation

All antibodies used in the study were bought commercially, and all commercially antibodies have been validated by the manufacturer as stated on their websites. Manufacturers state the antibodies have been validated for intended uses. Manufacturer citations are listed in manufacturer websites for each specific antibody.
Websites of the manufacturers were as follow: anti-PTEN antibody (138G6) (https://www.cellsignal.com/products/primary-antibodies/pten-138g6-rabbit-mab/9559), anti-eIF2α (D7D3) (https://www.cellsignal.com/products/primary-antibodies/eif2a-d7d3-xp-rabbit-mab/5324?_=1626092316657&Ntt=eif2%20d7d3&tahead=true), antibody to phosphorylated eIF2α (Ser51) (119A11) (https://www.cellsignal.com/products/primary-antibodies/phospho-eif2a-ser51-119a11-rabbit-mab/3597?_=1626092372386&Ntt=119a11&tahead=true), anti-GAPDH (1C4) (http://www.sungenebiotech.com/index.php?m=Product&a=product_xq&catid=2&proid=53&prid=290&pid=720&id=1557), anti-GFP (9F6) (http://www.sungenebiotech.com/index.php?m=Product&a=product_xq&catid=2&proid=54&prid=293&pid=704&id=1521), anti-FLAG (M2) (https://www.sigmaaldrich.cn/CN/en/product/sigma/f3165?context=product), anti-α-tubulin (2F9) (http://www.mbl-chinawide.cn/search-details2?id=1042&table=RuoAntibody), anti-puromycin (17H1) (https://www.sigmaaldrich.cn/CN/en/product/mm/mabe341?context=product), anti-GPX4 (E-12) (https://www.scbt.com/p/gpx-4-antibody-e-12?requestFrom=search), anti-LCMV (M104) (https://www.scbt.com/p/lcmv-antibody-m104?requestFrom=search), anti-Hsp90 (OTI4C10) (http://www.zsbio.com/product/TA-12), anti-N-PTEN (EPR23729-4) (https://www.abcam.com/pten-antibody-epr23729-4-ab260011.html), anti-HMGB1 (EPR3507) (https://www.abcam.com/hmgb1-antibody-epr3507-ab79823.html), anti-Hsp70 (EPR16892) (https://www.abcam.com/hsp70-antibody-epr16892-ab181606.html), anti-IL-17A (eBio17B7) (https://www.thermofisher.com/antibody/product/IL-17A-Antibody-clone-eBio17B7-Monoclonal/12-7177-81), anti-IL-10 (JES5-16E3) (https://www.thermofisher.com/antibody/product/IL-10-Antibody-clone-JES5-16E3-Monoclonal/12-7101-41), anti-Foxp3 (FJK-16s) (https://www.thermofisher.com/antibody/product/FOXP3-Antibody-clone-FJK-16s-Monoclonal/17-5773-82), anti-PD-1 (J43) (https://www.thermofisher.com/antibody/product/CD279-PD-1-Antibody-clone-J43-Monoclonal/46-9985-82), anti-TIM3 (RMT3-23) (https://www.thermofisher.com/antibody/product/CD366-TIM3-Antibody-clone-RMT3-23-Monoclonal/25-5870-82), anti-LAG3 (C9B7W) (https://www.thermofisher.com/antibody/product/CD223-LAG-3-Antibody-clone-eBioC9B7W-C9B7W-Monoclonal/17-2231-82), anti-CD45 (30-F11) (https://www.thermofisher.com/antibody/product/CD45-Antibody-clone-30-F11-Monoclonal/25-0451-81), anti-CD25 (PC61.5) (https://www.thermofisher.com/antibody/product/CD25-Antibody-clone-PC61-5-Monoclonal/12-0251-83), antibody to IFN-γ (XMG1.2) (https://www.thermofisher.com/antibody/product/IFN-gamma-Antibody-clone-XMG1-2-Monoclonal/17-7311-82), antibody to TNF-α (MP6-XT22) (https://www.thermofisher.com/antibody/product/TNF-alpha-Antibody-clone-MP6-XT22-Monoclonal/25-7321-82), anti-CD4 (GK1.5) (https://tonbobio.com/products/fitc-anti-mouse-cd4-gk1-5), anti-CD8 (53-6.7) (https://tonbobio.com/products/fitc-anti-mouse-cd8a-53-6-7 and https://tonbobio.com/products/apc-anti-mouse-cd8a-53-6-7), anti-G3BP1 (A14836) (https://abclonal.com.cn/catalog/A14836), anti-CD3ε (145-2C11) (https://www.biolegend.com/en-us/products/purified-anti-mouse-cd3epsilon-antibody-28), anti-CD28 (37.51) (https://www.biolegend.com/en-us/products/purified-anti-mouse-cd28-antibody-117), anti-IL-4 (11B11) (https://www.biolegend.com/en-us/products/purified-anti-mouse-il-4-antibody-894), anti-IFNγ (XMG1.2) (https://www.biolegend.com/en-us/products/purified-anti-mouse-ifn-gamma-antibody-998).

Eukaryotic cell lines

Policy information about cell lines

Cell line source(s)

B16-F10, HEK-293T, Molt4, Jurkat, CT26 and HeLa cells were from American Type Culture Collection (ATCC).

Authentication

None of the cell lines used were authenticated.

Mycoplasma contamination

All the cell line in this study were negative for mycoplasma contamination.

Commonly misidentified lines
(See ICLAC register)

No commonly misidentified cell lines were used.

Animals and other organisms

Policy information about studies involving animals; ARRIVE guidelines recommended for reporting animal research

Laboratory animals

Ptenα–/– mice (C57BL/6J background) were generated as described. Balb/c and Balb/c nude mice were purchased from Charles River Laboratories. 6-8 weeks old male mice were used for the study. The mice were reared in standard conditions with controlled temperature (20-26 خ), humidity (40-70%) and 12/12-hour dark/light cycle.

Wild animals

No wild animals were used.

Field-collected samples

No field-collected samples were used in the study.

Ethics oversight

All animal experimental protocols were approved by the Ethics Committee of Peking University Health Science Center.

Note that full information on the approval of the study protocol must also be provided in the manuscript.

Flow Cytometry

Plots

Confirm that:

- [x] The axis labels state the marker and fluorochrome used (e.g. CD4-FITC).
- [x] The axis scales are clearly visible. Include numbers along axes only for bottom left plot of group (a 'group' is an analysis of identical markers).
- [x] All plots are contour plots with outliers or pseudocolor plots.
- [x] A numerical value for number of cells or percentage (with statistics) is provided.

Methodology

Sample preparation	Lymphocytes in spleen were isolated by grinding the tissues in PBS containing 1% (vol/vol) FBS, followed by filtering through a 75 μM strainer. To isolate lymphocytes infiltrating in the lung, minced tissues were digested with 0.5 mg/ml Collagenase D (Roche) and 25 μg/ml DNase I (Sigma) at 37℃ for 30 mins. Then the tissues were grinded, and filtered through a 75 μM strainer. A 40% (10 ml)/70% (5ml) Percoll gradient (GE Healthcare) was used to isolate the tissues by centrifuging 800 g for 20 mins. Cells at the inter-layer were collected and counted for further operation. For isolation of tumor-infiltrating lymphocytes (TIL), tumors were minced, grinded and filtered like lungs mentioned above. Then the cells were resuspended in 5 ml FACS buffer, adding to a 40% (5 ml)/80% (5 ml) Percoll gradient. The gradient was centrifuged at 400 g for 45 mins, and the cells at the inter-layer of 40%/80% Percoll gradient were collected for further operation.
Instrument	FACSVerse, BD Biosciences.
Software	Flow cytometry data was collected using the FACSuite Software Bundle v1.0 (BD Biosciences) or FACSDiva software v6.1 (BD Biosciences). Flow cytometry data was analyzed using the FlowJo v7.6.1 software.
Cell population abundance	Murine CD8+ T cells were enriched using the MagniSortTMPositice Selection Beads (eBioscience) and the purity of sorted cells was ＞ 90% (determined by flow cytometry) .
Gating strategy	FSC/SSC plot indicating cell size and granularity were used to identify live lymphocytes. Boundaries between positive and negative were indicated by gates based on the isotype control staining. Expression of indicated proteins were evaluated on these populations as indicated in the figures and figure legends.

- [x] Tick this box to confirm that a figure exemplifying the gating strategy is provided in the Supplementary Information.
